# The relationship between health professionals perceptions of innovative work behavior and their metaverse knowledge and awareness levels

**DOI:** 10.1186/s12913-025-12492-4

**Published:** 2025-03-04

**Authors:** Tuba Duzcu

**Affiliations:** School of Health Sciences, Istanbul Medipol University, Istanbul, Turkey

**Keywords:** Innovative work behavior, Metaverse, Health professionals, Health management

## Abstract

**Background:**

Healthcare institutions have been affected by the changing environmental conditions with digitalization, and have turned to developing business models compatible with technological changes and adapting their institutions to these changes. For this change and adaptation, it is necessary to determine the innovative work behavior perception of healthcare professionals. This study aims to examine the relationship between healthcare professionals’ innovative work behavior perceptions and metaverse knowledge and awareness levels together with demographic characteristics.

**Methods:**

In the study, a quantitative research method was applied using the Metaverse (MS) Scale and Innovative Work Behavior (IWB) Scale, and the subject was examined with structural equation modeling. An online questionnaire prepared via Google Forms was applied to 253 healthcare professionals university graduated residing in Istanbul who accepted to participate in the survey voluntarily through snowball sampling. The responses obtained from the participants in the study were analyzed with SPSS for Windows 29.0 and AMOS 25.0 package programs. Discriminant validity analysis was performed between the variables and it was investigated whether the separation between the variables was sufficient for structural equation modeling. The effect of demographic characteristics on the IWB scale and the effect of IWB scale sub-dimensions on the MS scale sub-dimensions were investigated.

**Results:**

It was determined that the effect of IWB Scale sub-dimensions on MS Scale sub-dimensions was significant. The effects of the IWB Scale sub-dimensions “Opportunity Exploration” and “Idea Generation” on the MS Scale sub-dimensions “Technology, Digitalization, Social and Lifestyle” were found to be positive and significant. The effect of demographic characteristics such as “education, income, years of professional experience” on the sub-dimensions of the scales was found to be positive and significant.

**Conclusions:**

Today, it is an important requirement for healthcare professionals to have metaverse awareness and innovative work behavior perception. It is thought that it is important for healthcare managers and policy makers to measure these two levels of healthcare professionals and to include people with high levels of innovative work behavior perception and metaverse awareness in their institutions. In this study, healthcare professionals’ perception of innovative work behavior increases or affects the level of metaverse knowledge.

## Introduction

With the transition to Industry 4.0 in the world and especially after the Covid-19 Pandemic, increasing digitalization shows itself by making it possible to apply the latest technologies in healthcare services with Health 4.0 [1]. In this way, a higher quality healthcare service will be provided and all resources, including cost and human resources, will be used effectively and efficiently [2]. Since healthcare services play a very important role in the growth of countries and even the global economy, technology is an important factor in providing better healthcare services [3]. Health institutions, which are aware of this, have started to use metaverse technologies in general, namely “Extended reality (XR), Virtual Reality (VR), Augmented Reality (AR), Mixed reality (MR), Artificial intelligence (AI), User interaction (human-computer interaction), Robotics, machine learning (ML) and the internet of things (IoT), blockchain, Computer Vision, Network, Edge cloud applications, etc.” [4]. Virtual reality, the most popular of these, has actually been used in the fields of military and space studies for a long time. Today, together with other metaverse technologies, it is used in areas such as education, library, museum, game, industrial design as well as health. Metaverse, which is thought to shape the future of health, is currently used in areas such as education, research, rehabilitation, decision support system, clinical applications [5]. When articles on the use of metaverse in health in recent years are examined according to scientific fields, it is seen that the most studies are conducted in the field of “Psychology/Mental Health/Psychiatry”, followed by “Education/Digital Learning, Digital Transformation in Healthcare Services”, and the most used is “Virtual Reality/Rehabilitation”, followed by “Metaverse General and Augmented Reality” technologies [6]. Metaverse is expected to make potential improvements in many areas such as “treatment effectiveness, cost, healthcare workforce, education and patient satisfaction” in healthcare services. The use of metaverse technologies, which have a very important place especially in healthcare workforce planning, by healthcare professionals contributes to the development of application and clinical decision-making skills; It can also be used to perform critical tasks, prepare for extraordinary situations, or develop existing skills and competencies [5, 7].

In today’s world where there is intensive information production and consumption, data has become more valuable than money; digitalization, artificial intelligence and metaverse technologies have entered our lives; in the process of economic, social and societal systems adapting to the age, it is now a necessity for institutions to use metaverse technologies, which are of vital importance for growth, more actively [3, 8]. For this reason, it is important to measure the knowledge and awareness levels of healthcare professionals about the metaverse. Healthcare institutions, which have different characteristics compared to other institutions and even conduct education and research activities in university hospitals, dynamic environments and variable work environments, direct their employees to adapt to new generation learning processes by following developments in education and technology in order to maintain their existence in a rapidly changing world. Rapid changes in technology in healthcare and all areas, increasing customer expectations, the necessity to keep up with continuous innovation, have put pressure on institutions to respond to the problems encountered quickly, creatively and ethically, making it necessary for them to focus on innovation and use current technologies. Healthcare institutions have also been affected by this situation and have turned to developing business models compatible with technological changes, and to rapidly adapt or develop their processes, products and services in order to increase efficiency, ensure sustainability and be successful [9]. Therefore, it is important for healthcare institution managers to have this vision [10]. For this change and adaptation, it is necessary to measure healthcare workers’ perception of innovative work behavior. Innovative work behavior is of vital importance for the growth and development of any social or economic sector worldwide and has developed as a valuable concept that changes the efficiency of healthcare practitioners [11]. It is stated that IWB “involves adopting targeted techniques that allow employees to successfully implement new ideas and adjust operational strategies to improve results” [12]. Since there is no comprehensive quantitative study in the literature measuring healthcare workers’ perception of metaverse knowledge and awareness, and in order to fill the gap in this area, this study aimed to examine the relationship between healthcare workers’ perception of innovative work behavior and the level of metaverse knowledge and awareness together with demographic characteristics.

### Metaverse in health

Today, as the internet is increasingly entering our lives, the time spent online is increasing day by day in Turkiye as well as in the world. The Dijtal 2024 report shows that “the time spent using the internet in Turkiye is 6 hours and 57 minutes, 17 minutes above the world average and 20th in the world ranking” [13]. In addition, according to the Turkish Statistical Institute (TUIK) Household Information Technologies Usage Survey, 2024 report, “The internet usage rate in Turkiye was 87.1% in 2023 for individuals aged 16–74, while it was 88.8% in 2024, and the internet usage rate was 92.2% for men and 85.4% for women” [14]. Although the Metaverse seems like a very new concept, it is a concept that emerged in 1995. This concept, whose use is increasing day by day and an increasing trend in its use is seen in 2021, shows itself especially with studies based on computer science and engineering; Today, it is also seen in fields such as social sciences, business and management [3]. In Turkiye, the concept of the metaverse has attracted the attention of researchers from many different fields including religion, fashion, marketing, tourism, economy, health and sports [15]. The concept of the metaverse first rose in 2021 after the pandemic and increased rapidly after the name of Facebook was changed to “Meta” in 2021, as seen in Google Trends 2023 search data [16]. It is possible to say that this concept in health has gained momentum especially as of 2023 [6]. The concept of “Metaverse in Health”, which gained serious momentum in June-July 2024, continued to gain momentum in 2024 [17].

There is no widely accepted definition for the concept of metaverse. It can be said that “A metaverse could be a mix of virtual reality (VR) system and augmented reality (AR) in real-world contexts where people can engage themselves in discussions, collaborations, or learning from experiences”. The digital economy, due to its many features such as the ability of multiple users to work together in real time in virtual environments, real-time audio, video interaction, and information sharing, etc., has begun to play a role in the digital transformation in healthcare in recent years [1, 18, 19]. The metaverse in healthcare began with the participation of Seoul in 2021. Thanks to the “feeling of being there” provided by the metaverse, the opportunities provided by the metaverse contribute to the provision of healthcare services, especially in countries with a small number of medical personnel and those trying to ensure geographical equity by caring for patients at long distances [3]. It is thought that metaverse technologies, which provide many benefits such as improved patient care, interdisciplinary collaboration, reduction of treatment costs, increase in quality, improvement of patient outcomes, monitoring of patient recovery, increase in patient compliance, reliability in operations, reduction of errors, creation of a decision support system, increase in efficiency, decrease in workload, decrease in time loss, contribution to education, will be used more in the future [1, 19–21].

Metaverse awareness has been measured in different sectors with different scales, and when the literature was examined, no metaverse scale specific to the health sector was found. In this study, the Metaverse Scale [7], which was developed in Turkiye, its reliability and validity were performed, and is used to measure metaverse awareness in the national and international health sector, was used [20, 22]. The scale has four sub-dimensions: *“Technology (TC)*,* Digitalization (DG)*,* Social (SC) and Lifestyle (LS)”*.

### Innovative work behavior in health

The concept of innovative work behavior is intertwined with innovation and transformation. In institutions where there are innovative leaders and a modern management approach prevails, creativity should be used to create new ideas and employees should be able to implement these new ideas. It is necessary to state the importance of the emergence and development of new ideas not by pressure but by the employees’ own will and the adoption of this behavior in the formation of this concept. Thus, as Janssen [23] stated, the concept of innovative work behavior, which is a complex behavior consisting of these three behavioral tasks, emerges [8, 9]. Messmann & Mulder [24] express innovative work behavior as a concept that includes “seeing, noticing and evaluating new thoughts and ideas, creating an action strategy and providing support for innovative ideas” [8]. Kanter (1988) stated that employees have four individual tasks in the innovation process. These tasks are “Idea Generation, Coalition Building, Idea Realization and Transfer/Diffusion” [25]. According to Jong & Hartog (2008), innovative work behavior factors include four dimensions: “Opportunity Exploration, Generation of ideas, Fighting for ideas, Implementation of ideas” [26]. In the article in which the Innovative Work Behavior scale used in this study was developed, five multi-dimensional dimensions were put forward as “Opportunity Exploration, Idea Generation, Idea Promotion, Idea Realization (differentiated in two sub-dimensions: Criterion-based implementation and learning-based communication) and Idea sustainability (differentiated in two sub-dimensions: External dissemination and internal embedding)”. In this study, the dimensions “Opportunity Exploration (OE) and Idea Generation (IG)” that overlap with the purpose of the study were included in the study. The hypotheses of the study were created by considering these two dimensions included in the study together with the Metaverse sub-dimensions and sociodemographic characteristics. “Messmann (2012) defines IWB as a multi-stage iterative process, consisting of four different phases: Opportunity Exploration which entails paying attention to trends, opportunities for innovation and problem recognition; Idea Generation, i.e. generating novel and useful ideas for products, services or processes” [25].

Although it is seen in the literature that many scales have been developed regarding innovative work behavior [23, 27–36], the scale developed by Lambriex-Schmitz et al. [25] in 2020 and adapted to Turkish by Baş and Balaban [9] in 2021 and whose reliability and validity studies were conducted in Turkiye was used in this study because it is a current and widely used scale.

In the healthcare sector, where digitalization is rapidly increasing, being able to keep up with change and adopt an innovative approach will be possible with the support of healthcare managers, both individually and institutionally. Therefore, this study aims to measure healthcare professionals’ perception of innovative work life behavior with a current scale and to clarify a topic not encountered in the literature by examining the relationship of this perception with the level of metaverse knowledge and awareness. Hypotheses were created to investigate the effect of OE, INC, which are sub-dimensions of IWB, and income level, which is a sociodemographic characteristic, on Metaverse sub-dimensions TC, DG, SC, LS. In addition, a hypothesis was created regarding the effect of sociodemographic characteristics on IWB. All hypotheses are stated under a separate heading in the Methodology section.

## Methodology

### Study design

In this study, in order to examine the relationship between healthcare professionals’ innovative work behavior perceptions and metaverse knowledge and awareness levels together with demographic characteristics, quantitative research method was applied using two different scales available in the international index and the subject was examined with structural equation modeling.

### Hypothesis

The hypotheses created in line with the aims and objectives of the study, together with the sub-dimensions of the scales and demographic characteristics in line with the information provided in the conceptual framework, are given below:

- H#1: Sociodemographic characteristics of healthcare professionals have a statistically significant effect on their perceptions of innovative work behavior.
- H#2: Income level of healthcare professionals has a statistically significant effect on their perceptions of innovative work behavior.
- H#3: Income level of healthcare professionals has an effect on their level of metaverse knowledge and awareness.
- H#4: Healthcare professionals have an effect on the technology dimension of opportunity exploration.
- H#5: Healthcare professionals have an effect on the digitalization dimension of opportunity exploration.
- H#6: Healthcare professionals have an effect on the social dimension of opportunity exploration.
- H#7: Healthcare professionals have an effect on the lifestyle dimension of opportunity exploration.
- H#8: Healthcare professionals have an effect on the technology dimension of idea generation.
- H#9: Healthcare professionals have an effect on the digitalization dimension of idea generation.
- H#10: Healthcare professionals have an effect on the social dimension of idea generation.
- H#11: Healthcare workers have an impact on the lifestyle dimension of idea generation.

### Partipicants

The universe of the study consists of healthcare professionals over the age of 23 who are university graduates living in Istanbul/Turkiye. Since the number of healthcare workers per city in Turkiye is not published and this information is not accessible, numerical information about the universe was not shared. Istanbul was chosen because it is the largest city in Turkiye, has the highest population, has the most healthcare institutions, is a city where health tourism is carried out and has the most healthcare professionals. It is not possible to reach all healthcare professionals living in Istanbul. Data were collected from healthcare professionals who met the criteria (residing in Istanbul, over 23 years of age, university graduates) from two foundation university hospitals and one public hospital. It was determined that approximately 710 people met these criteria. Since the healthcare professionals in the sample were healthcare professionals with an associate degree or higher, participants aged 23 and above were included in the study. The reason for the sample to consist of university graduates is that the IWB scale used as a data collection tool is recommended to be used in university-level samples [25]. The sample size was calculated according to Bartlett et al. [37]. The formula used for sample size is the formula known as the sample size calculation formula with a known population size. N = universe size, n = sample size, S = standard deviation (0.5), d = deviation amount tolerance level (0.05) and t: standard table value for 95% confidence level, the sample size calculation formula to be selected from a universe with a known population size is as follows [37];

$$n=\frac{n_0}{1+{\displaystyle\frac{n_0}N}}\;and\;n_0=\left[\left(t.S\right)/d\right]^2$$

The population size was estimated to be 710 and above and found as $\:{n}_{0}=\left[\right(\text{1,96.0,5})/{\text{0,05}]}^{2}$ =384. When this value was substituted into the sample number formula, the sample size was calculated as; $\:n=\frac{384}{1+\frac{384}{710}}$ = 249. When the invalid surveys were removed, a total of 253 valid surveys were reached.

The online survey prepared via Google Forms was applied to 253 healthcare professionals over the age of 23 (Doctor/Nurse/Midwife, Employees in administrative services, Health Technician/Technician/Biologist and Other Health Workers) residing in Istanbul who accepted to participate in the survey voluntarily through snowball sampling.

We found it appropriate to use snowball sampling to determine people who are more relevant to the subject. The data collection process was terminated considering that this study was a cross-sectional study, was conducted using the snowball sampling method, sample saturation was reached, and extending the process further would cause it to lose its cross-sectional nature. According to Bayram (2010), a sample size between 200 and 400 is sufficient for structural equation modeling. In this study, in order to select university graduate healthcare professionals over the age of 23 and not to lose the cross-sectionality of the study due to the reasons mentioned before, the data collection phase was terminated after the desired saturation was reached and invalid surveys were excluded from the study.

### Data collection

In the questionnaire form prepared according to 5 and 6-point Likert scale, a total of 10 questions were included in the first section regarding sociodemographic characteristics and internet and metaverse usage. In the second section, two dimensions (OE, IG) of the *“Innovative Work Behavior Scale”* [25] and the Scale Adapted to Turkish [9] were used and in the third section, the *“Metaverse Knowledge and Awareness Scale”* [7]. The *“Innovative Work Behavior Scale”* is a 6-point Likert-type scale consisting of 2 dimensions and a total of 11 items. It consists of the items Opportunity Exploration (OE) (Q1,Q2,Q3,Q4) and Idea Generation (IG) (Q5,Q6,Q7,Q8,Q9,Q10,Q11). The is a 5-point Likert-type scale consisting of 4 dimensions and a total of 15 questions. These dimensions and their items are as follows: Technology (Q1,Q2,Q3,Q4,Q5,Q6,Q7), Digitalization (Q8,Q9,Q10), Social (Q11,Q12), Lifestyle (Q13,Q14,Q15). Responses to items in the MS scale, which has been validated in Turkiye, are rated on a five-point Likert-type scale ranging from 1 = strongly disagree to 5 = strongly agree. The average score of a subscale is calculated by dividing the scores given to all items by the number of items. The higher the score from a subscale, the higher the level of what is measured by that subscale. The level of knowledge, attitude and awareness of participants about the Metaverse increases as the scale scores increase. The lowest total score that can be obtained from the scale is 15, and the highest total score is 75 [7]. Data were collected from participants who agreed to participate in the survey between June and October 2024.

### Ethical approval

The survey form created was analyzed by conducting a pilot study and approval numbered E-10840098-202.3.02–2972 was received from Istanbul Medipol University Non-Interventional Clinical Research Ethics Committee for the final version of the survey form on 16.05.2024. Approval was received from the participants for the first question of the survey and information was provided with the statement “The data to be collected in the study will be evaluated only within the scope of the research and will not be transferred to third parties under any circumstances.” In addition, in order to avoid conceptual confusion; an explanation of the Metaverse concept and information about the applications/technologies within its scope were provided in the explanation section before the questions in the form. Informed consent to participate was obtained from all of the participants in the study.

### Data analysis

#### Methods used in data analysis

The responses obtained from 253 participants in the study were analyzed with SPSS for Windows 29.0 and AMOS 25.0 package programs. In the frequency analysis of the sample, demographic characteristics and descriptive information about working life were presented with percentage rates. Confirmatory factor analyses of the Innovative Work Behaviour Scale and Metaverse Scale included in the questionnaire form were conducted and the consistency, validity and reliability in the sample were measured by calculating Cronbach’s alpha, Composite reliability and Average Variance Explained (AVE) values. Discriminant validity analysis was conducted between the variables to investigate whether the separation between the variables was sufficient for structural equation modeling. The effect of demographic characteristics (INC: Income, EDU: Education level, GND: Gender, AGE: Age, YPW: Year of professional work) on the Innovative Work Behaviour scale and the effect of the Innovative Work Behaviour scale sub-dimensions on the Metaverse scale sub-dimensions were investigated.

## Results

### Demographic characteristics distribution

The gender distribution of the participants is 90.5% female and 9.5% male. 72.7% of our participants are single, while 27.3% are married. In the age groups, the 23–28 age group is 72.7%, the 29–34 age group is 13.0%, the 35–40 age group is 7.9%, the 41–46 age group is 4%, and the 47 and above age group is 2.4%. The educational backgrounds of the participants in the sample are 74.7% associate degree, 17.4% bachelor’s degree, 7.5% master’s degree, and 0.4% doctorate degree. The income of 65% of the participants is between 17.001 and 22.000 Turkish Lira (TL), and 15.4% is between 22.001 and 27.000 TL (Table 1).

**Table 1:** Percentage distribution of demographic characteristics of the sample

		*n*	%
Gender	Female	229	90.5%
	Male	24	9.5%
Age	23–28	184	72.7%
	29–34	33	13.0%
	35–40	20	7.9%
	41–46	10	4.0%
	47 and above	6	2.4%
Your Marital Status	Single	184	72.7%
	Married	69	27.3%
	Associate Degree	189	74.7%
Educational Status	Bachelor’s Degree	44	17.4%
	Master’s Degree	19	7.5%
	Doctorate	1	0.4%
	17.001–22.000	166	65.6%
Monthly Net Income (Turkish Lira-TL)	22.001–27.000	39	15.4%
	27.001–32.000	19	7.5%
	32.001–37.000	9	3.6%
	37.001–42.000	4	1.6%
	42.001 and above	16	6.3%

The sample includes 0–3 years of experience groups as 57.3%, 4–7 years as 23.3%, 8–11 years as 8.7%, and 12 years and above as 10.7%. It is seen that 0.8% of the participants are physicians, 33.6% are nurses/midwives, 46.2% are employees in administrative services, 33.6% are other health workers, and 15.4% are Health Technicians/Technicians/Biologists. It is understood that 7.5% spend less than 1 h on the internet, 46.2% 1–3 h, 29.6% 3–5 h, and 16.6% 5 h and above. 11.5% of the participants stated that they had an experience with the metaverse. It is seen that the participant group included 11.9% foundation university employees, 81.0% private hospital employees, 4.3% city hospital employees, and 2.8% training and research hospital employees (Table 2).

**Table 2:** Distribution of occupational characteristics of individuals in the sample

		*n*	%
Your Profession	Physician	2	0.8%
	Nurse/Midwife	10	4.0%
	Employees in administrative services	117	46.2%
	Health Technician/Biologist Technician/Technician/Biologist	39	15.4%
	Other Health Worker	85	33.6%
Year of professional work	0–3	145	57.3%
	4–7	59	23.3%
	8–11	22	8.7%
	12 and above	27	10.7%
Time Spent on the Internet in a Day	Less than 1 h	19	7.5%
	Up to 1–3 h	117	46.2%
	Up to 3–5 h	75	29.6%
	5 h and Above	42	16.6%
Have You Experienced Metaverse Technologies?	Yes	29	11.5%
	No	224	88.5%
Type of Hospital You Work	Foundation University Hospital	30	11.9%
	Private Hospital	205	81.0%
	City Hospital	11	4.3%
	Training and Research	7	2.8%

In the significance test of the scale sub-dimensions according to the time spent on the internet, no significant difference was found in all sub-dimensions. (*p* < 0.05) The mean values of both scale sub-dimensions did not differ according to the time spent on the internet (Table 3).

**Table 3:** Significance test of scale sub-dimensions according to time spent on the internet

		*N*	Mean	SD	KW	*p*
Opportunity Exploration	Less than 1 h	19	4.3553	1.18531	3.853	0.278
	Up to 1–3 h	117	4.7842	0.71386		
	Up to 3–5 h	75	4.7533	0.82864		
	5 h and Above	42	4.8750	0.89927		
	Total	253	4.7579	0.82642		
Idea Generation	Less than 1 h	19	4.4286	1.08379	5.39	0.145
	Up to 1–3 h	117	4.9096	0.74764		
	Up to 3–5 h	75	4.9810	0.90208		
	5 h and Above	42	4.8741	0.94297		
	Total	253	4.8888	0.86231		
Technology	Less than 1 h	19	4.2105	1.03495	4.816	0.186
	Up to 1–3 h	117	4.3785	0.54634		
	Up to 3–5 h	75	4.4381	0.97071		
	5 h and Above	42	4.5102	0.91834		
	Total	253	4.4054	0.79572		
Digitalization	Less than 1 h	19	4.1404	1.06177	2.969	0.396
	Up to 1–3 h	117	4.4302	0.61753		
	Up to 3–5 h	75	4.3422	0.99996		
	5 h and Above	42	4.5714	0.89044		
	Total	253	4.4058	0.83070		
Social	Less than 1 h	19	3.6842	1.37649	5.021	0.17
	Up to 1–3 h	117	4.2607	0.79762		
	Up to 3–5 h	75	4.1733	1.15517		
	5 h and Above	42	4.3452	1.17124		
	Total	253	4.2055	1.03378		
Lifestyle	Less than 1 h	19	4.2105	1.10112	0.901	0.825
	Up to 1–3 h	117	4.4615	0.68488		
	Up to 3–5 h	75	4.2978	1.03334		
	5 h and Above	42	4.3413	1.06811		
	Total	253	4.3742	0.89954		

### Confirmatory factor analyses of scales included in the model

In the confirmatory factor analysis, as the sample size increases, the Chi-Square (x^2^) calculated parameter is found to be high in samples higher than 200 and the Chi-Square (x^2^) test statistic is found to be insignificant [38–40]. In the confirmatory factor analysis of the scales included in the study, it was decided whether the model was within the desired ranges or not by examining the Chi-Square (x^2^) value (x^2^/sd) adjusted with the degree of freedom and other fit index values and the parameters present in the standardized residual covariance matrix [41] (Table 4).

**Table 4:** Index ranges and concordance coefficients considered in confirmatory factor analysis [42]

Indexes	Good Fit	Acceptable Fit	IWB	MVS
*x*^2^ / df	0 ≤ χ2/df ≤ 2	2 < χ2/df ≤ 3	1.801	2.987
GFI	≥ 0.90	0.85–0.89	0.908	0.911
CFI	≥ 0.95	≥ 0.90	0.943	0.940
SRMR	≤ 0.05	0.06 ≤ SRMR ≤ 0.08	0.044	0.076
RMSEA	≤ 0.05	0.06 ≤ RMSEA ≤ 0.08	0.056	0.069

### Confirmatory factor analysis for innovative work behavior scale (IWB)

In the confirmatory factor analysis applied to Innovative Work Behavior Scale, which is in the literature with its 11-item and two-dimensional structure, since the factor loadings were (FL > 0.50) for all 11 items, no items were excluded from the analysis. Confirmatory factor analysis was concluded with 11 items in two dimensions as in the literature. In the analysis, the factor loading standard values are in the range of (0.65; 0.92). When the model parameters in Table 4 are taken into consideration, Innovative Work Behavior Scale is within the limits of “acceptable fit”.

### Confirmatory factor analysis for metaverse scale (MVS)

In the confirmatory factor analysis applied to Metaverse Scale (MVS), which is in the literature with 15 items and 4 dimensions, since all of the items were (FL > 0.5), no items were eliminated from the analysis. In the analysis, it is understood that the factor loading standard values are in the range of (0.61; 0.94). According to the model indexes in Table 4, it is understood that the Metaverse Scale is within the “acceptable fit” limits.

### Convergent and discriminant validity applied to variables included in the model

The composite reliability (CR) coefficient is calculated from the standard loadings found in the confirmatory factor analysis. If the composite reliability coefficient is found as (CR ≥ 0.70), it can be stated that the “composite reliability condition” is met [43].

Convergent validity is the average variance explained (AVE) coefficient. The fact that the convergent validity condition is met is checked by this value being (AVE ≥ 0.50). If the entire composite reliability value is found as (CR ≥ 0.70), it is also acceptable to find (AVE ≥ 0.40). The discriminant validity condition is checked by the condition that the value calculated by taking the square root of the (AVE) coefficient is higher than the correlation coefficient in the relevant row and column [39].

The reliability value calculated from all items of Innovative Work Behavior Scale was determined as (0.939) and the reliability value calculated from all items of Metaverse Scale was determined as (0.934). It is seen that “high reliability” level was obtained for both scales. Innovative Work Behavior scale sub-dimensions were found as Opportunity Exploration (0.912) and Idea Generation (0.89). The sub-dimensions of Idea Generation scale were determined as Technology (0.911), Digitalization (0.901), Social (0.929), and Lifestyle (0.910). “High reliability” degree was obtained for all dimensions in the model. Since the value found from all scales in the combined reliability values ​​was (CR ≥ 0.70), the combined reliability condition was met. Since the average explained variance coefficients were (AVE ≥ 0.50), the convergent validity condition was also met. Since the square root value of the AVE coefficients is higher than the correlation coefficients in the relevant row and column, the discriminant validity condition is also met. In the Metaverse scale, the general mean of the scale was calculated as 3.802 ± 0.66 and the means of its sub-dimensions were determined as DG dimension 4.01, TC dimension 3.86, SC dimension 3.69, and LS dimension 3.65. The highest total score obtained from the scale was 66.79, while the lowest score was 27.43. In the Innovative Work Behaviour scale, the general mean was 4.82 ± 0.69 and the means of its sub-dimensions were IG dimension 4.89, OE dimension 4.76. The highest total score calculated from the scale was 59.45 and the lowest score was 17.53 (Table 5).

**Table 5:** Convergent and discriminant validity values ​​calculated from standard factor loadings

**Scale**	**Dimension**	**Mean**	**SD**	**OE**	**IG**	**TC**	**DG**	**SC**	**LS**
**IWB**	**OE**	4.76	0.83	**(0.781)**					
**IWB**	**IG**	4.89	0.86	0.661^**^	**(0.806)**				
**MS**	**TC**	3.86	0.75	0.367^**^	0.387^**^	**(0.837)**			
**MS**	**DG**	4.01	0.65	0.366^**^	0.362^**^	0.565^**^	**(0.802)**		
**MS**	**SC**	3.69	0.56	0.312^**^	0.360^**^	0.748^**^	0.536^**^	**(0.794)**	
**MS**	**LS**	3.65	0.70	0.380^**^	0.327^**^	0.522^**^	0.509^**^	0.371^**^	**(0.801)**
	Cronbach’s Alpha (CA)			0.912	0.890	0.911	0.901	0.929	0.910
	Composite reliability (CR)			0.919	0.909	0.921	0.905	0.924	0.918
	Average Variance Extracted (AVE). (AVE)			0.611	0.654	0.701	0.644	0.632	0.643

*OE* Opportunity Exploration, *IG* Idea Generation, *TC* Technology, *DG* Digitalization, *SC* Social, *LS* Lifestyle, *Mean* Arithmetic mean, *SD* Standard deviation

^***^p < 0.001

^**^p < 0.01

^*^p < 0.05

### Structural equation modeling path analysis applied to observed values of the research model

**Fig. 1:**
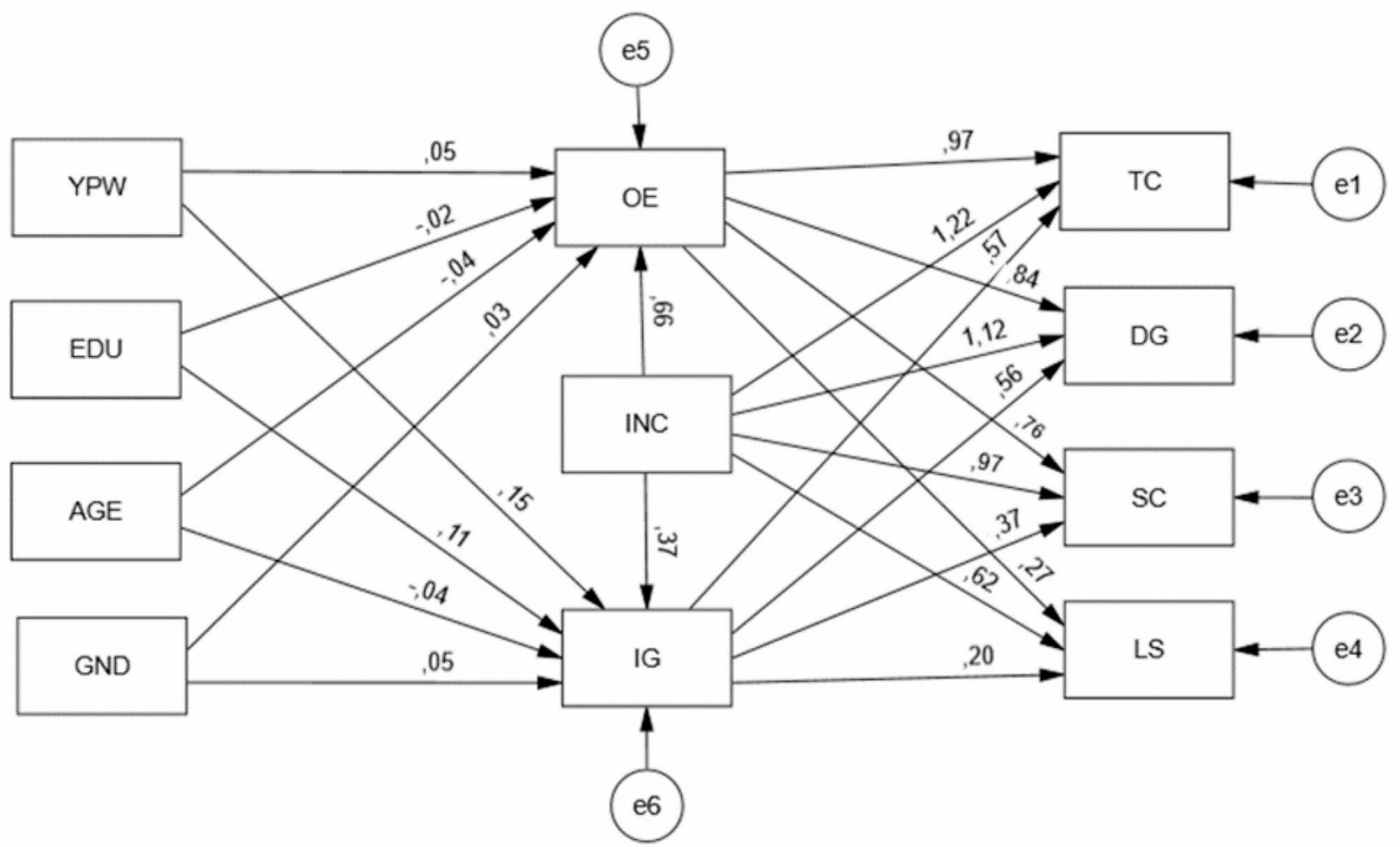
AMOS graphic structure of path analysis model with calculated variables

The research model in Fig. 1 was tested as a path analysis model with observed variables calculated using AMOS program 25.0. In the estimation of model parameters, the “Asymptotically Distribution-free (ADF)” parameter estimation method, which is asymptotically independent of distribution, was preferred. This method can be used if some of the variables are measured with an ordinal scale and the others are continuous or if the distributions of continuous variables deviate significantly from normality or if the variables examined are dichotomous [44].

In the research model, the effects of the demographic characteristics “Year of professional work, education, age, gender and income” variables on the Opportunity Exploration and Idea Generation Digital dimension, and the effects of the “income variable” and the Opportunity Exploration and Idea Generation Digital variables on the Technology, Digitalization, Social and Lifestyle variables were examined. Since income is more general information than other demographic characteristics, it was included in the model in this way. In other words, since the participants who work in the same health institution but have different incomes are not in the same group, income is considered to be a more significant and determining feature, and this path was followed (Fig. 1).

The model is significant since x^2^ (70.540), x^2^/df (2.613) are calculated as model indices (*p* < 0.05) in the path analysis model with the calculated variables. Since the model’s fit indices GFI (0.906), CFI (0.932), SRMR (0.0753), RMSEA (0.06750) are within the acceptable fit range, it is understood that the model is valid. The model regression parameters are presented in (Table 6).

**Table 6:** Significance test of direct effect regression coefficients in the model

Independent		Dependent	Estimate	Std. Estimate	Z	*P*	Hypothesis
EDU	➔	OE	− 0.022	− 0.024	− 0.450	0.653	Reject
GND	➔	IG	0.132	0.049	1.055	0.292	Reject
YPW	➔	IG	0.096	0.151	1.998	0.048^*^	Accept
EDU	➔	IG	0.118	0.115	1.956	0.050^*^	Accept
AGE	➔	OE	− 0.027	− 0.041	− 0.557	0.578	Reject
GND	➔	OE	0.084	0.034	0.706	0.480	Reject
AGE	➔	IG	− 0.028	− 0.038	− 0.431	0.666	Reject
YPW	➔	OE	0.031	0.052	0.632	0.527	Reject
INC	➔	OE	0.329	0.663	4.976	***	Accept
INC	➔	IG	0.198	0.366	2.789	0.005^**^	Accept
OE	➔	LS	0.385	0.271	2.066	0.039^*^	Accept
OE	➔	DG	1.016	0.837	4.665	***	Accept
IG	➔	SC	0.524	0.369	3.238	0.001^**^	Accept
OE	➔	SC	1.179	0.762	4.699	***	Accept
IG	➔	DG	0.624	0.561	4.860	***	Accept
OE	➔	TC	1.124	0.968	4.680	***	Accept
IG	➔	LS	0.259	0.199	2.251	0.024^*^	Accept
INC	➔	DG	0.677	1.125	4.409	***	Accept
INC	➔	SC	0.741	0.966	4.396	***	Accept
INC	➔	LS	0.437	0.622	3.625	***	Accept
INC	➔	TC	0.701	1.217	4.246	***	Accept
IG	➔	TC	0.608	0.571	4.411	***	Accept

*OE* Opportunity Exploration, *IG* Idea Generation, *TC* Technology, *DG* Digitalization, *SC* Social, *LS* Lifestyle, *INC* Income, *EDU* Education level, *GND* Gender, *YPW* Year of professional work

^*^p < 0.05

^**^p < 0.01

^***^p < 0.001

In Table 5, where the direct regression effects in the path analysis model with the calculated variables are examined, it was determined that the effect of the Innovative Work Behaviour Scale sub-dimensions on the Metaverse Scale sub-dimensions was significant (*p* < 0.05). The effect of the income (INC) variable on the Innovative Work Behaviour Scale sub-dimensions and the Metaverse Scale sub-dimensions was also significant (*p* < 0.05). The effect of the Education status variable (EDU) on the Idea Generation (IG) variable and the effect of the Years of Work (YPW) variable on the Opportunity Exploration (OE) variable among the demographic variables were found to be significant (*p* < 0.05). It was observed that there was no significance in the other direct effect hypotheses (*p* > 0.05) (Table 7). Accordingly;

**Table 7:** Summary hypothesis acceptance/rejection table

Hypothesis Number	Independent		Dependent	*P*	Hypothesis Acceptance/Rejection
H#1	EDU	➔	OE	0.653	Reject
H#1	GND	➔	IG	0.292	Reject
H#1	YPW	➔	IG	0.048^*^	Accept
H#1	EDU	➔	IG	0.050^*^	Accept
H#1	AGE	➔	OE	0.578	Reject
H#1	GND	➔	OE	0.480	Reject
H#1	AGE	➔	IG	0.666	Reject
H#1	YPW	➔	OE	0.527	Reject
H#2	INC	➔	OE	***	Accept
H#2	INC	➔	IG	0.005^**^	Accept
H#3	INC	➔	DG	***	Accept
H#3	INC	➔	SC	***	Accept
H#3	INC	➔	LS	***	Accept
H#3	INC	➔	TC	***	Accept
H#4	OE	➔	TC	***	Accept
H#5	OE	➔	DG	***	Accept
H#6	OE	➔	SC	***	Accept
H#7	OE	➔	LS	0.039^*^	Accept
H#8	IG	➔	TC	***	Accept
H#9	IG	➔	DG	***	Accept
H#10	IG	➔	SC	0.001^**^	Accept
H#11	IG	➔	LS	0.024^*^	Accept

- The effect of the Opportunity Exploration variable on the Technology variable (β = 0.968; *p* < 0.05) was found to be positive and significant, the effect on the Digitalization variable (β = 0.837; *p* < 0.05) was found to be positive and significant, the effect on the Social variable (β = 0.762; *p* < 0.05) was found to be positive and significant, and the effect on the Lifestyle variable (β = 0.271; *p* < 0.05) was found to be positive and significant.
- The effect of the Idea Generation variable on the Technology variable (β = 0.571; *p* < 0.05) was found to be positive and significant, the effect on the Digitalization variable (β = 0.561; *p* < 0.05) was found to be positive and significant, the effect on the Social variable (β = 0.369; *p* < 0.05) was found to be positive and significant, and the effect on the Lifestyle variable (β = 0.199; *p* < 0.05) was found to be positive and significant.
- The effect of the income variable on the Opportunity Exploration variable (β = 0.663; *p* < 0.05) was found to be positive and significant, the effect on the Idea Generation variable (β = 0.366; *p* < 0.05) was found to be positive and significant, the effect on the Technology variable (β = 1.217; *p* < 0.05) was found to be positive and significant, the effect on the Digitalization variable (β = 1.125; *p* < 0.05) was found to be positive and significant, the effect on the Social variable (β = 0.966; *p* < 0.05) was found to be positive and significant, and the effect on the Lifestyle variable (β = 0.622; *p* < 0.05) was found to be positive and significant.
- The effect of the education level (EDU) variable on the Idea Generation variable (β = 0.115; *p* < 0.05) was found to be positive and significant, and the effect of the years of professional experience (YPW) variable on the Idea Generation variable (β = 0.151; *p* < 0.05) was found to be positive and significant.

## Discussion

This study aimed to examine the relationship between healthcare professionals’ innovative work behavior perceptions and metaverse knowledge and awareness levels together with demographic characteristics. In the study, it was determined that the Innovative Work Behavior Scale sub-dimensions had a significant effect on the Metaverse Scale sub-dimensions. The effect of demographic characteristics such as “education, income, year of professional work” on the sub-dimensions of the scales was found to be positive and significant.

### Metaverse scale

In the Metaverse scale, the general mean of the scale was calculated as (3.802 ± 0.66) and the means of its sub-dimensions were determined as DG dimension 4.01, TC dimension 3.86, SC dimension 3.69, and LS dimension 3.65. Similarly, in the study of Işık and Üstünakı [5], the mean scores of the sub-dimensions were TC (22.8 ± 6.04), DG (8.8 ± 3.48), LF (10.3 ± 3.27), SC (6.6 ± 2.34), respectively. In Demir’s [16] study, unlike this study, the sub-dimension with the highest mean was the LS dimension. After this, similar to this study, there are DG, TC and SC sub-dimensions. Ergin et al. In the study [20], the participants obtained the highest mean score from the LS dimension (3.96 ± 0.70), the lowest mean score from the DG sub-dimension (3.49 ± 0.81), and the mean score of the scale is (3.74 ± 0.56), similar to the one in this study. In general, it is possible to say that the participants’ level of metaverse awareness is good. It is thought that the high level of awareness, especially in a group with a high young age range, despite the lack of this experience, is an interesting result and that the participants’ perspective on these technologies is positive.

There are studies in the literature using the scale used in this study regarding MS awareness. According to Süleymanoğulları et al. [7], who developed the MS scale; the highest score that can be obtained from the scale is 75. The highest score obtained from the MS scale in this study is 66.79, and the lowest score is 27.43. This shows that the MS awareness level of the participants in the study is high. A higher Metaverse total score mean (48.5 ± 12.45) is found compared to other articles [5].

Although there are studies conducted with patients and students on metaverse (MS) awareness; in general, no study has been found in healthcare professionals where metaverse awareness was studied with another variable [3, 20]. In the study conducted by Ergin et al. [20], which specifically aimed to measure nurses’ metaverse knowledge, attitude and awareness, it was determined that 46.2% of the time spent on the internet was 1–3 h. In this study, the time spent on the internet was predominantly 1–3 h and it was observed that the average values ​​of the scale sub-dimensions did not differ according to the time spent on the internet. In another study, a significant difference was found only in the DG sub-dimension, and it was observed that “participants who used the internet for 2–4 hours had a higher average than participants who used the internet for less than 2 hours” [16]. In the study of Ergin et al. [20], a weak, positive correlation was found between the scores obtained from the TC, DG and LF sub-dimensions of the daily internet use variable.

In this study, the effect of the income variable (INC) on all dimensions of the MS scale was found to be positive and significant. The hypothesis *“H#3: Income level of healthcare professionals has an effect on their level of metaverse knowledge and awareness.”* was accepted. In the literature, it has been observed that “no significant difference was detected in the digitalization, social and lifestyle sub-dimensions, only a significant difference was detected in the technology sub-dimension” in the income variable [16]. Income appears as a factor affecting the perception of innovative behavior and Metaverse awareness. In this study, where the majority of participants have an income level of minimum wage and above in Turkiye, it is thought that it is possible that there was no experience with the Metaverse (88.5%). People with higher income levels can more easily acquire digitalization-related equipment. Therefore, it is natural that income levels affect metaverse experience and awareness. It is noteworthy that income also affects IWB. It has been observed that participants with higher salaries, such as managers, have a higher perception of innovative work behavior. It is possible to comment that the perspectives of those working with low salaries are only about doing the given job and continuing the process, and that they do not make any effort to bring any innovation to their work. In this sense, it is possible to say that income can be a tool that can be used to increase perspectives on innovative work behavior and new technologies such as the metaverse.

The effect of the Opportunity Exploration variable on all MS scale sub-dimensions (TC, DG, SC, LS) was found to be positive and significant (*H#4*,* H#5*,* H#6*,* H#7 were accepted).* The effect of the Idea Generation variable on all MS scale sub-dimensions (TC, DG, SC, LS) was found to be positive and significant *(H#8*,* H#9*,* H#10*,* H#11 were accepted)*. In other words, the effect of all sub-dimensions of the IWB scale on all sub-dimensions of the MS scale was positive and significant. In this case, the high perception of innovative work behavior of the participants included in the study will also increase the level of Metaverse knowledge and awareness. The fact that the sample consists mostly of healthcare managers (46.2%) who are in the administrative part of the job and medical secretaries, patient consultants, etc. who manage patient-institution relations in the field shows that institutions can make decisions about adapting to digital technologies quickly and with the right steps. The fact that the management in healthcare institutions has a high awareness of innovative work behavior, adopts the trends towards digitalization and using new technologies and has a positive view on its use is of great importance in change management and digitalization studies. In a study where digital health services were evaluated by hospital administrators, it was determined that “digital health services are very valuable for all units, especially intensive care units, and it is estimated that digitalization in healthcare services will continue in both private and public hospitals” [45]. Studies conducted with nurses also revealed that healthcare professionals have positive perceptions in this direction, that they have heard of the concept of metaverse before and are familiar with it, and that they believe that these technologies can be used for patient care and virtual healthcare service delivery [20]. In this study, other healthcare professionals and health technicians/biologists constitute the other dominant groups, respectively, and their perspectives on digitalization in the field are positive. According to the literature, it can be said that healthcare professionals who are further away from the management pyramid are less sensitive in change management, while management is more willing [46]. Contrary to this situation, the willingness of healthcare professionals outside the management level to be involved in the process is important in terms of increasing the success of healthcare institutions in change management and digitalization. In order to create such an environment, managers who are visionary, adopt innovative work behavior and digital technologies, and have a modern management philosophy should be present in health institutions. With the activities that these managers will plan and carry out to adapt their employees to innovations, employees will be motivated and will minimize the problems caused by external factors affecting the institution by keeping up with the modern age. In short, managers will be able to increase this awareness indirectly by increasing employees’ perceptions of innovative work behavior rather than just carrying out activities aimed at this concept in order to increase metaverse awareness. Some studies can be done to increase this perception. However, it would be beneficial for human resources managers to evaluate sociodemographic characteristics such as educational status (EDU) and years of professional experience (YPW) as some of the important criteria in recruitment. In this way, there will be an increase in IWB and MS awareness directly and indirectly.

### IWB scale

There are studies in the literature where innovative work behavior of healthcare workers is studied with other variables; however, no study has been found investigating its relationship with Metaverse awareness. In a study where the IWB scale used in this study was used and the participants were nurse managers; it was determined that 42.9% of the participants exhibited highly innovative work behavior and 49.2% exhibited moderate innovation [47]. In his study with healthcare workers, Palumbo [46] determined the positive effects of employees’ busyness on innovative behaviors and revealed that a positive organizational climate creates an empowering work environment that further encourages innovation. In a study examining healthcare workers’ ethical climate perception and innovative work behavior, it was observed that “there is a statistically significant and positive relationship between the two variables” [48]. In this study, where two dimensions of the IWB scale were included, when the mean, standard deviation and *p* values of OE and IG were examined (OE Mean: 4.76- Standard Deviation: 0.83- p: 0.278_IG Mean: 4.89- Standard Deviation: 0.86-p: 0.145), it is possible to say that the level of innovative work behavior is at a good level.

*H#1: Sociodemographic characteristics of healthcare workers have a statistically significant effect on their perceptions of innovative work behavior.*

In this hypothesis, the effects of educational status (EDU) and years of professional experience (YPW) were found to be significant only on the IG sub-dimension, while other characteristics were not found to be significant. This hypothesis can generally be described as irrefutable.

In the literature, it was also observed that there is no significant difference in the IWB sub-dimensions in terms of gender [9]. On the other hand, another study revealed that the personal characteristics of nurse managers do not have a statistically significant relationship with innovative work behaviors [47].

In this study, the effect of the education level variable (EDU) on the Idea Generation (IG) variable was found to be significant. It is possible to say that as the education level increases, the emergence of new ideas also increases. However, contrary to this study, a study conducted with nurse managers indicated that the education level had no significant relationship with any stage of IWB. However, it was stated that this result could have been reached because nurses with the same education level were included in the study mentioned [47]. Although all participants are university graduates, this result was reached when evaluated according to associate degree, bachelor’s degree, master’s degree and doctorate levels.

In this study, the effect of the income variable (INC) on all dimensions of the IWB scale was found to be positive and significant. It can be said that the income level is also a reflection of the education level of healthcare professionals. Therefore, as in the case of education, income appears as a distinct and determining feature for participants who work in the same institution but are not in the same group. Accordingly, participants with higher incomes will internalize the stages of opportunity search and new idea generation more in order to contribute more to the institution. In this case, it is possible to say that the hypothesis *“H#2: The income level of healthcare professionals has a statistically significant effect on their perception of innovative work behavior”* is accepted.

In this study, the effect of the Years of Professional Experience (YPW) variable on the Idea Generation (IG) variable and the Opportunity Exploration (OE) variable was found to be positive and significant. As professional experience increases, the perception of innovative work behavior will also develop in order to advance in one’s career, improve oneself, provide better healthcare services and contribute to the institution. For these purposes, healthcare professionals will be more willing to search for opportunities and generate new ideas. Lambriex-Schmitz et al. As stated by [25], innovative behavior will enable employees to participate in management, thus empowering staff and thus becoming an important component of institutional success. It is thought that this behavior can lead to positive outcomes such as improving patient outcomes, increasing the quality of healthcare service delivery and patient satisfaction, and thus increasing the success and profitability of the healthcare institution [49].

Table 7 presents a summary of all hypotheses along with their dimensions and sub-dimensions. It can be said that all of the hypotheses put forward in this study are irrefutable. Because two of the features in the H#1 hypothesis, which includes sociodemographic characteristics, are accepted, it can be said that the H#1 hypothesis is irrefutable. All of the other hypotheses are accepted.

### Implication for health managerse, researchers and policymakers

It is possible to say that as the level of education increases, the emergence of new ideas also increases. For this reason, it is thought that it would be beneficial to work with people with a high level of education, especially in areas such as change management, production of new projects, adaptation to innovation, research and use of new technologies, where new ideas are desired to be produced or new ideas are desired to be presented.

The effect of the income variable on all dimensions in the IWB and MS scales was found to be positive and significant. Therefore, as the income level increases, the perception of innovative work behavior will increase and MS awareness this rate will again reflect MS awareness positively. It is possible to say that the fair distribution of income level is important and that this information should be taken into consideration in determining the income levels of people in studies and projects where the objectives are especially digitalization, change management, etc. Including participants with high professional experience years in these studies will contribute to the process in the opportunity research and idea generation stages.

Particularly, the high perception of innovative work behavior of the participants in these projects will ensure that MS awareness is also high. For this reason, it is thought that it would be useful to measure the IWB perception levels of the people involved in the process with a series of tests in the selection of the people involved in the process or in the recruitment process in these studies conducted institutionally. The literature also supports this information. For example, Korku’s [50] study with health managers showed that the authentic leadership skills of health managers affect innovative work behavior. Ergin at al [20]’s study aimed to measure the metaverse knowledge, attitude and awareness of nurses; It was revealed that 81.6% of the nurses believed that they could provide patient education using the metaverse in the future, and 46% believed that they could do virtual nursing. Mohamed et al. [47] stated that nurse managers should empower their staff and create a positive work environment to encourage innovative behavior.

In this context, for health managers, politicians and health workers; It is recommended that in-service training should include training to encourage innovative behavior, integrated communication applications that enable departments to speak the same language within the organization should be used and coordination should be ensured in this way, technological infrastructure should be strengthened and information should be shared effectively and efficiently with intra-organizational intranet etc. applications, an organizational climate should be created that empowers employees and ensures their participation in management, evidence-based scientific applications should be increased, innovations in health and management science should be followed, current information and technologies should be used in health and management science, the needs of the age should be correctly identified and the use of necessary digital applications should be adopted, applications that increase patient and employee compliance/satisfaction such as telemedicine and telesurgery should be adopted and used when appropriate for the institution [49].

### Limitations

In this study, the participants were only healthcare professionals aged 23 and over who were university graduates living in Istanbul. It is thought that future studies can be conducted with a larger comparative sample in different cities. This research is a cross-sectional study and the results cannot be generalized because of the limited sample size. The data is limited in scope as it was obtained through Google forms.

In addition, due to the density of healthcare professionals in Turkiye and their exposure to many surveys, there are difficulties and limitations in including them in the survey. These points and the inability to reach the entire universe are among the limitations of the study.

## Conclusions

The fact that there is no study in the literature examining the relationship between healthcare professionals’ innovative work behavior perceptions and metaverse knowledge and awareness levels together with demographic characteristics and using these variables together suggests that the study is original. It is considered important for healthcare professionals, health policy makers and academics working in this field to investigate these two issues, examine the relationship between the variables and evaluate the results by experts. This study also reveals the effect of innovative work behavior on metaverse awareness. Therefore, it is very important for healthcare professionals to be open to innovation, especially in areas where technology is more required, such as education, recruitment, training, treatment, etc., in short, to have high innovative work behaviors. In today’s world where paperless/digital hospitals are on the agenda and their numbers are increasing day by day; all healthcare managers should be careful to choose their teams from people who are highly perceptive not only in terms of digitalization but also in terms of the sub-dimensions of innovative behavior such as opportunity exploration and idea generation. Because, as revealed in this study, a high level of this perception will also increase metaverse awareness. In Turkiye and many other countries, hospitals want to take their hospitals to the next level by being accredited in terms of digitalization. In this way, they aim to prevent waste and errors and provide patient-centered and quality healthcare services. Change management is much better in hospitals accredited by institutions such as the Healthcare Information and Management Systems Society (HIMMS), and healthcare personnel or employees in this project team should be carefully selected in this regard. It is especially important for health managers in health institutions with such goals to act in this minimal manner. In tertiary healthcare institutions, which have duties such as education and research in addition to healthcare service delivery; health technologies are also used in health education. It is thought that the metaverse universe and virtual reality technology will have a positive impact on healthcare services in other areas where it will be used, such as diagnosis, treatment, surgery, etc. together with healthcare education. Success will be achieved with healthcare professionals who have a high perception of innovative behavior and aim to use digitalization to increase the quality of healthcare services and to contribute to healthcare professionals and healthcare institutions. Because the use of metaverse in healthcare is expected to offer potential opportunities and advantages in many areas such as health tourism, human resources, treatment effectiveness in healthcare services, education, patient satisfaction and data management. It is also thought that metaverse technologies can create an opportunity for countries that have difficulties in the amount of manpower in healthcare along with the mobility of healthcare personnel. In the literature, it is stated that the use of metaverse in healthcare services has benefits in terms of remote access, education, innovative treatments, data collection, collaboration, facilitating the work of employees, increasing information exchange in the digital environment, etc. Health managers and policy makers need to evaluate the advantages and disadvantages of metaverse technologies and make the right decisions and create strategies for micro and macro level applications. For this, it is thought that it is important for both themselves and their teams to have high perceptions of innovative work behavior and to select teams from people with high levels of innovative work behavior and metaverse awareness in such technologically substructured studies. It is aimed that this study contributes to the field and provides information and sheds light to academicians, healthcare managers and healthcare policy makers who want to conduct research and create strategies in this field.

## Data Availability

The datasets used and analysed during the current study are available from the corresponding author on reasonable request.
